# Ultrasonographic features of *Lucilia sericata*-induced wound myiasis with foot gas gangrene: A case report

**DOI:** 10.1016/j.radcr.2026.03.016

**Published:** 2026-04-11

**Authors:** Yuki Munekata, Tomoya Kawamura, Jumpei Nishikawa, Toshimasa Tennoji, Yuki Ogino, Hiroka Aonuma, Yuki Takahashi

**Affiliations:** a Department of Emergency Medicine, Sunagawa City Medical Centre, Sunagawa, Japan; b Department of Plastic and Reconstructive Surgery, Sunagawa City Medical Centre, Sunagawa, Japan; c Division of Clinical Laboratory, Sunagawa City Medical Centre, Sunagawa, Japan; d Department of Tropical Medicine, The Jikei University School of Medicine, Tokyo, Japan

**Keywords:** Myiasis, *Lucilia sericata*, Gas gangrene, Point-of-care ultrasonography, Color Doppler, Twinkle artefact

## Abstract

Myiasis is the tissue infestation by fly larvae, mainly *Dermatobia hominis* or *Lucilia sericata*. Sonographic features of *D. hominis* myiasis are well described but not those of *L. sericata* with concurrent gas gangrene. A 57-year-old man presented with a 1-month left foot injury, swelling, and functional decline. Examination revealed left foot gangrene with the loss of the fifth toe, maggot infestation, and systemic inflammation. Laboratory tests revealed diabetes mellitus. Point-of-care ultrasound showed subcutaneous air, fluid–debris layers, and hyperechoic, moving structures consistent with larvae with posterior acoustic shadowing, and cobblestone appearance. Although it is often difficult to distinguish larvae from gas, as both appear hyperechoic, colour Doppler imaging revealed an associated twinkle artefact, differentiating larvae from gas. Computed tomography confirmed gas gangrene. Above-knee amputation was performed. Wound cultures showed *Proteus mirabilis* and *Streptococcus dysgalactiae.* DNA analysis identified *L. sericata*. We report the ultrasonographic features of *L. sericata*-induced wound myiasis with gas gangrene, underscoring the diagnostic value of point-of-care colour Doppler imaging in complex wound infections. In malodorous wounds with intralesional gas, the presence of self-propelling movement and posterior acoustic shadow, combined with a twinkle artefact on colour Doppler imaging should raise suspicion for myiasis as an important differential diagnosis.

## Graphical abstract

**Figure:**
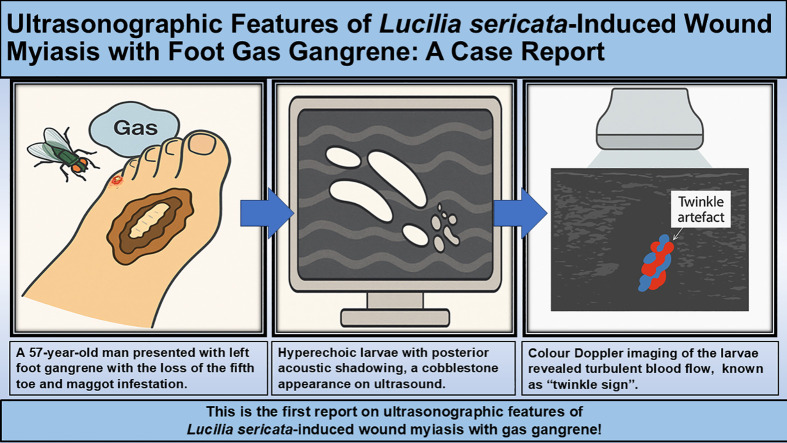

## Introduction

Myiasis is caused by tissue invasion of flies of the order Diptera (2-winged flies) [1]. Cutaneous myiasis is the most common type of myiasis, occurring at the site of egg penetration. It can be further divided into furuncular, wound, migratory, and cavitary myiasis. *Dermatobia hominis*, a human botfly, commonly causes furuncular cutaneous myiasis in a broad range of mammals [2,3]. The use of computed tomography (CT) for detecting larvae remains controversial, as this imaging modality often fails to demonstrate definitive larval findings, as illustrated in our case and previous case reports [3]. Moreover, CT scanning has several disadvantages, including radiation exposure and time consumption. In contrast, point-of-care ultrasound (POCUS) enables real-time dynamic assessment while reducing ionizing radiation exposure and improving cost-effectiveness [[4], [5]]. Several studies have described the use of ultrasonography for myiasis caused by *D. hominis*, including its sonographic characteristics [3,[6], [7], [8], [9], [10], [11], [12], [13], [14], [15], [16], [17], [18], [19]]. In particular, Doppler ultrasonography using a high-resolution soft-tissue transducer allows direct visualization of larvae, facilitating more rapid confirmation of myiasis with high sensitivity and specificity [7,14]. Myiasis is occasionally caused by *Lucilia sericata*, a fly belonging to the Calliphoridae family and a very common species in the temperate zone of the Northern Hemisphere [16]. To date, no case report has documented the sonographic features of wound myiasis caused by *L. sericata* in association with foot gas gangrene. Here, we present a case report detailing the ultrasonographic characteristics of *L. sericata*-induced wound myiasis.

## Case report

A 57-year-old man with a left foot injury was brought to the Department of Emergency Medicine at our institute. The patient reported that the injury had occurred approximately 1 month earlier when he had dropped an object on his left foot. However, he was unable to recall the exact nature of the object. Initially, the patient attempted to manage the injury himself. However, his condition subsequently deteriorated, and he became unwell and unable to ambulate properly. His medical history was unremarkable, with no known diagnosis of diabetes mellitus.

**Fig. 1:**
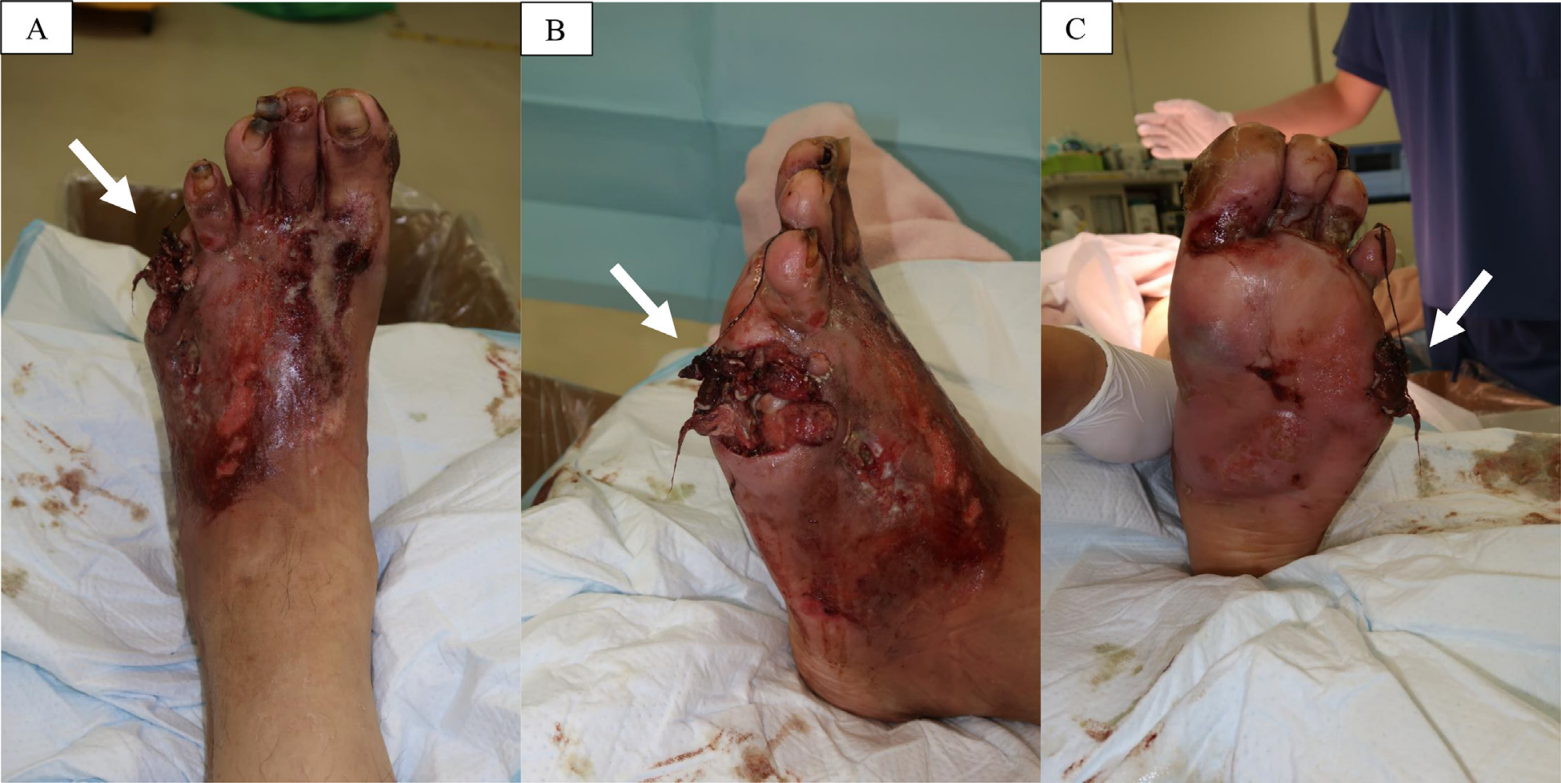
The patient’s left foot before the operation. (A-C) The left foot exhibited marked swelling and poor hygiene, with absence of the fifth toe exposing underlying bone. Multiple larvae were observed within the wound site (white arrow).

**Fig. 2:**
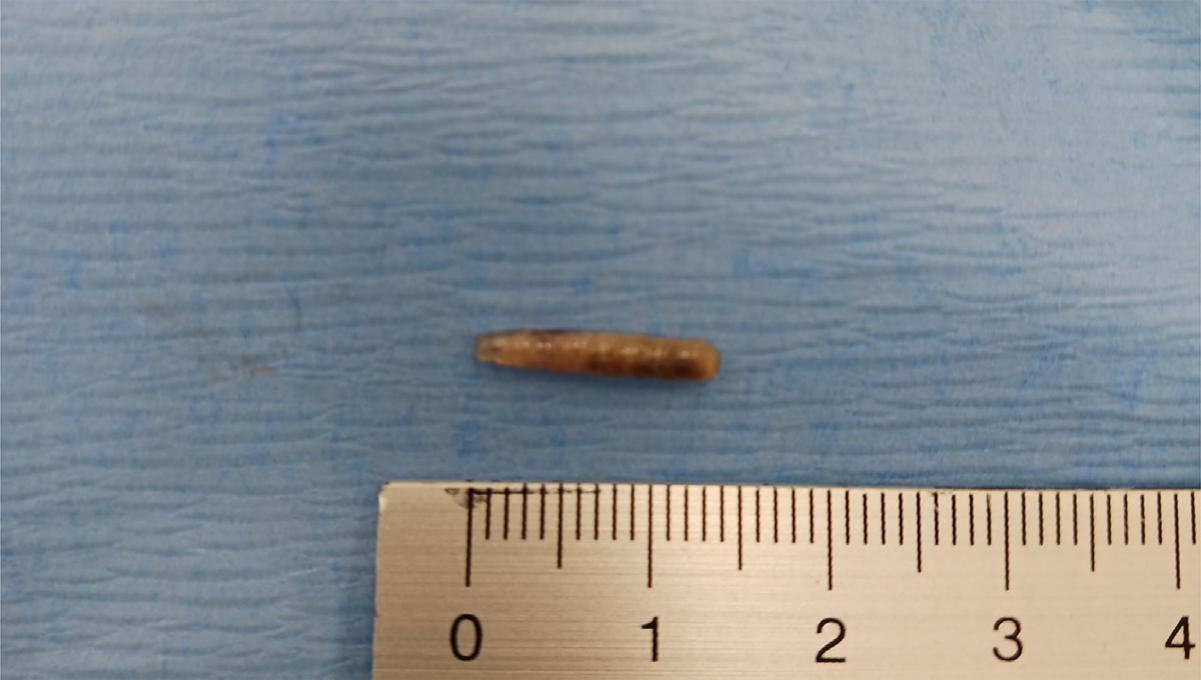
Macroscopic aspect of a larva. The average length of each larva ranged from 1.0 to 1.5 cm.

**Fig. 3:**
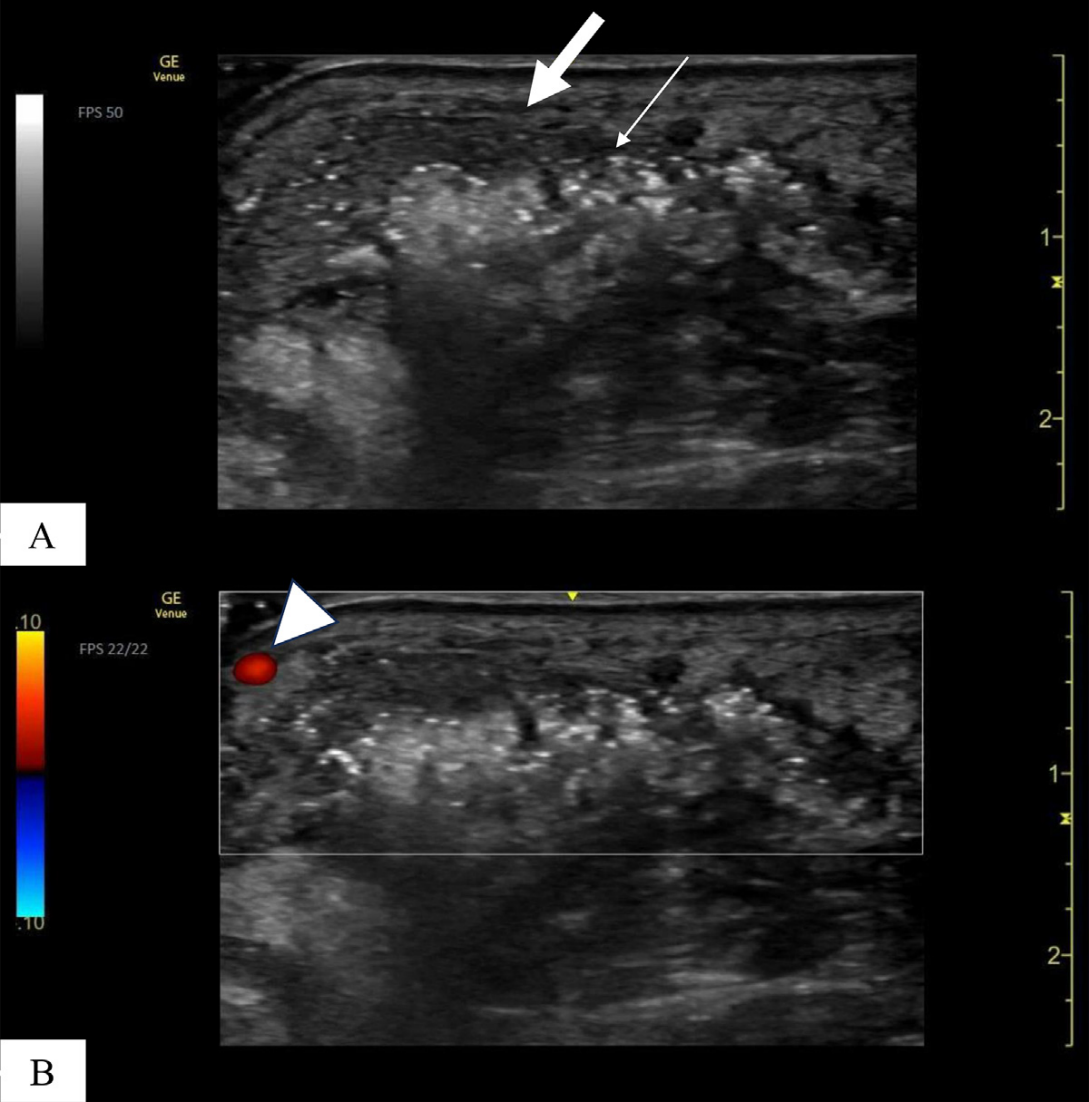
Ultrasound images of the injured site. (A) The subcutaneous layer demonstrated a thick hypoechoic region containing anechoic areas consistent with debris or fluid (thick white arrow), along with hyperechoic foci suggestive of intralesional gas (thin white arrow). (B) Colour Doppler imaging demonstrated the absence of intralesional flow within the subcutaneous abscess, while an adjacent blood vessel exhibited detectable Doppler flow (white arrowhead).

**Fig. 4:**
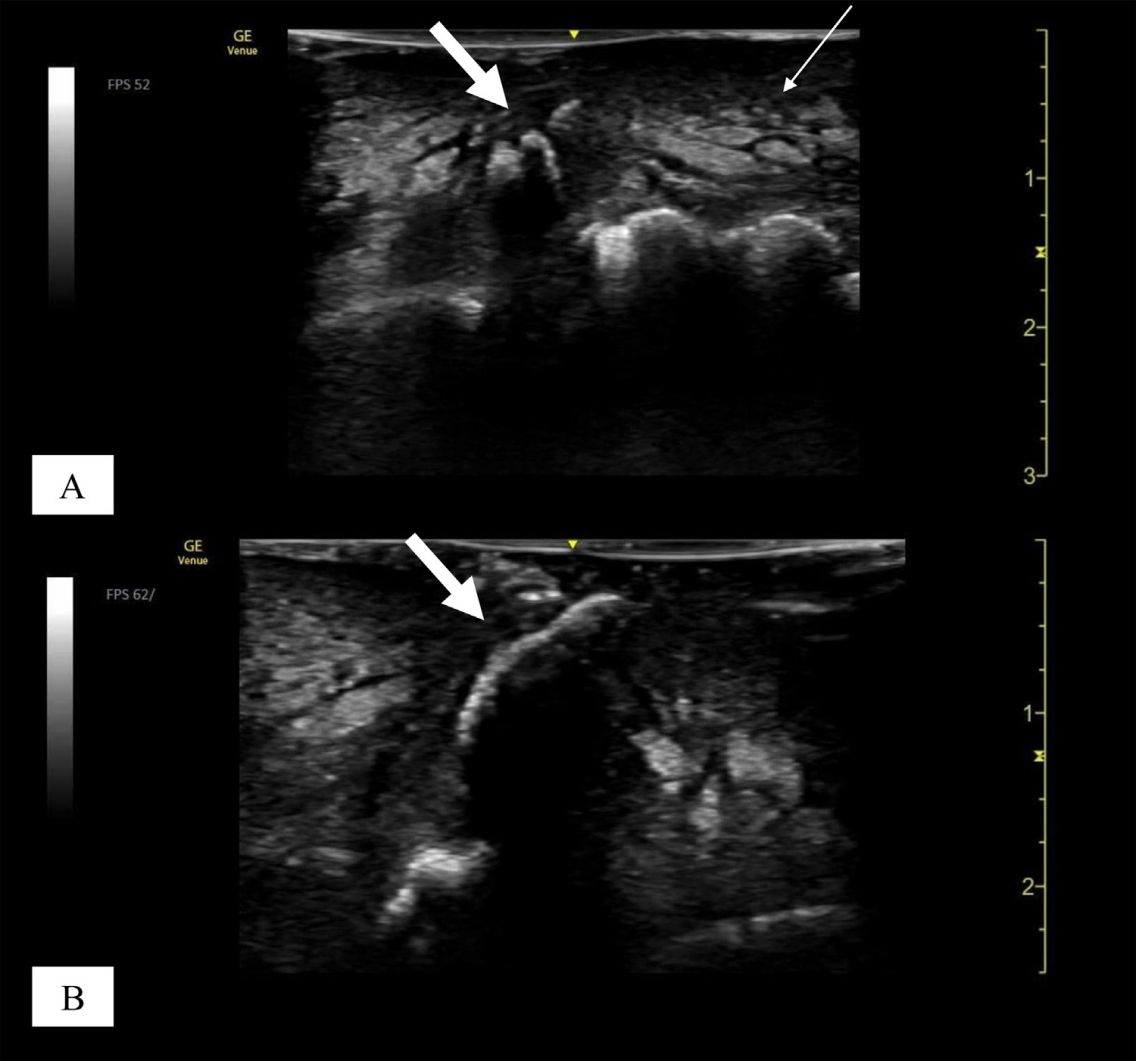
Ultrasound images of larvae. (A) Within the subcutaneous layer, hyperechoic structures with posterior acoustic shadowing were visualised, consistent with the presence of larvae (thick white arrow). Adjacent tissue exhibited a cobblestone appearance (thin white arrow). (B) A longitudinal view of a larva was visualised within the subcutaneous layer (thick white arrow).

**Fig. 5:**
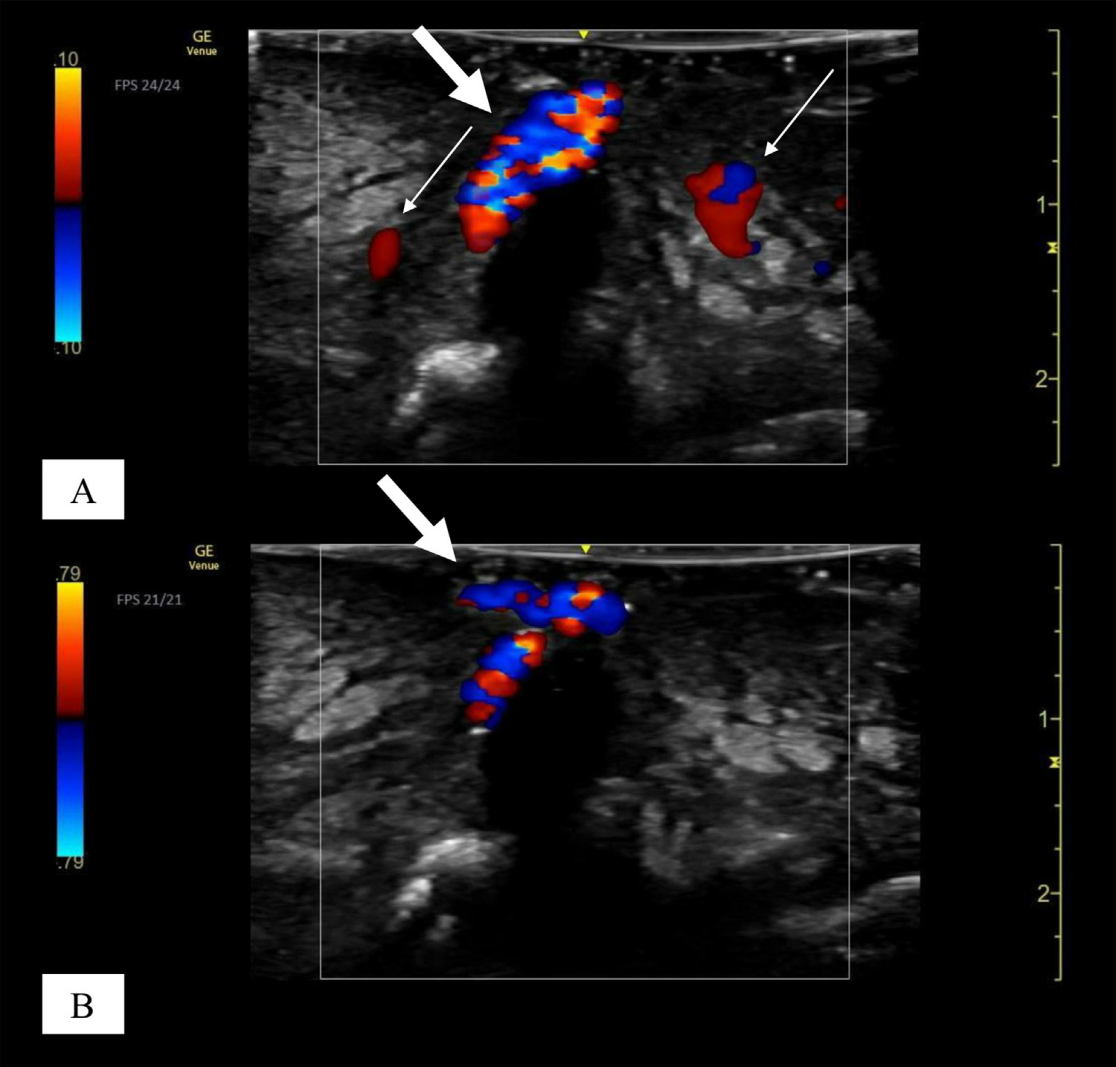
Colour Doppler images of larvae. (A) The larval structure exhibited a sonographic twinkle sign (thick white arrow), and blood vessels demonstrated flow signals (thin white arrow) at a Doppler setting of 10 cm/s. (B) At a setting of 79 cm/s, no vascular flow was detected, while the twinkle artefact persisted (thick white arrow).

**Fig. 6:**
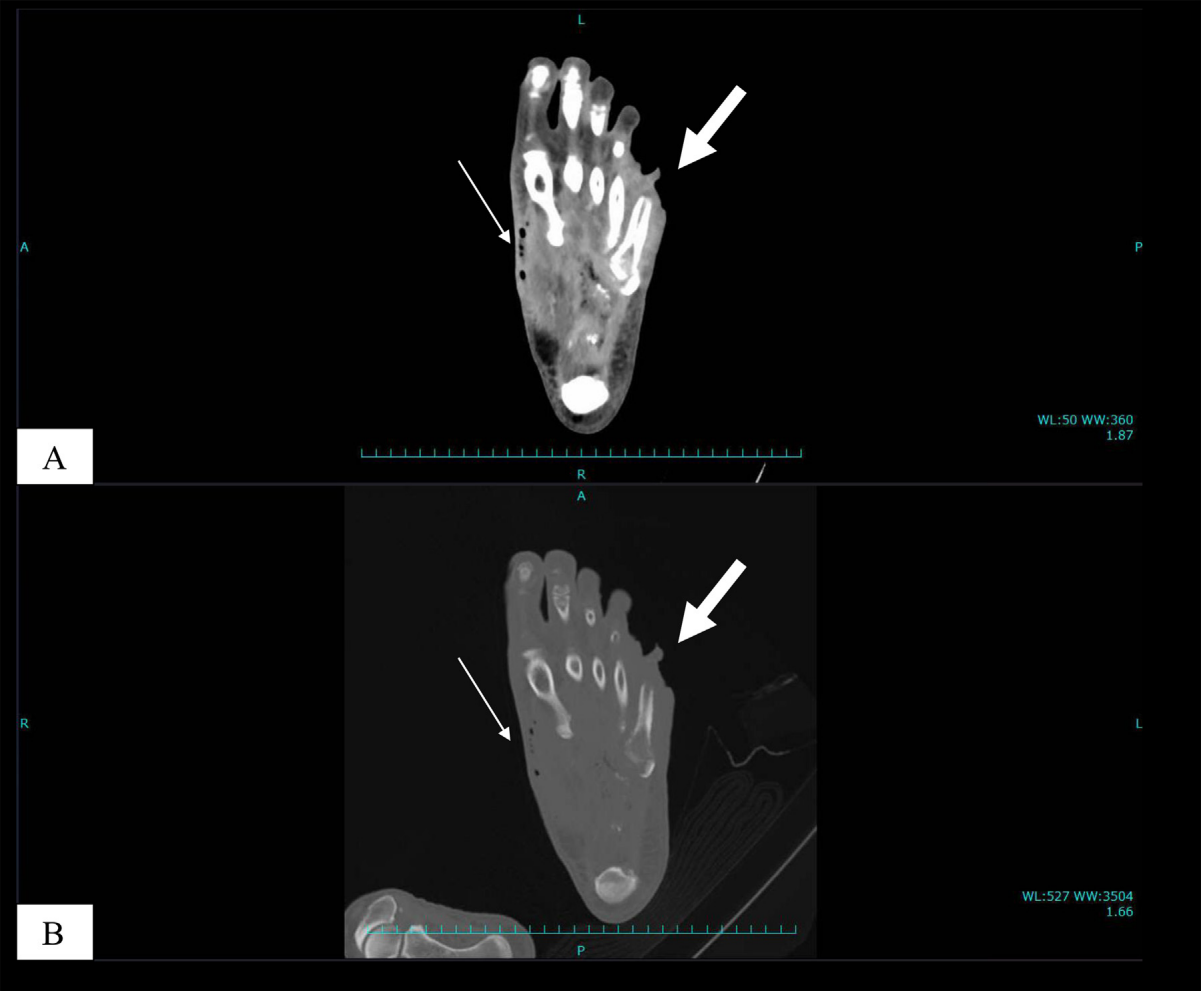
CT images of the injured site. (A & B) The proximal-distal phalangeal bones of the fifth toe show absent signals (thick white arrow). An abscess with gas was present in the plantar region (thin white arrow).

On physical examination, the patient appeared moderately unwell and presented with tachycardia and fever; however, he remained alert and oriented. Examination of the left foot revealed generalised swelling and poor hygiene, with the absence of the fifth toe. The affected area showed signs of gangrene and exudate, with the presence of maggots, which were viable and motile, indicating myiasis (Figs. 1 and 2). The patient reported the absence of pain in the affected area, accompanied by a mildly diminished tactile sensation. Laboratory investigations revealed leucocytosis of 21,700 cells/μL, a C-reactive protein level of 28.88 mg/dL, a serum lactate concentration of 6.2 mmol/L, a random blood glucose level of 249 mg/dL, and an HbA1c of 8.9%. A POCUS examination was also performed using a 4-12 MHz linear transducer that revealed the presence of a thick, complex subcutaneous layer with hyperechoic foci, suggestive of a subcutaneous abscess containing gas (Fig. 3). The thickened subcutaneous layer was easily compressible with the application of pressure, further supporting the presence of an abscess (Supplementary Video 1). In addition, hyperechogenic self-propelling structures were observed within the subcutaneous abscess, suggesting the presence of larvae beneath the skin (Fig. 4 and Supplementary Videos 2 and 3). With a velocity range of 10-79 cm/s, colour Doppler imaging of the larvae revealed constant alternating Doppler signals, which was consistent with a sonographic “twinkle sign” (Fig. 5, Supplementary Videos 4 and 5). Computed tomography revealed the absence of proximal-distal phalanges of the left fifth toe, along with the presence of an abscess and intralesional gas in the left foot, underpinning the diagnosis of gas gangrene. However, the presence of maggots could not be confirmed using CT imaging (Fig. 6). Moreover, 2 sets of blood cultures and a skin swab were obtained from the site of injury. Although both blood cultures yielded negative results, the skin swab culture confirmed the presence of *Proteus mirabilis* and *Streptococcus dysgalactiae*. Additionally, several maggots were collected from the wound site, and DNA analysis of the samples was performed. The results confirmed that the larvae were *L. sericata*.

After careful examination, the patient was urgently referred to plastic and reconstructive surgeons at our institute, and an emergency above-knee amputation of the left lower limb was performed. The surgery was performed without complications, and the patient was managed postoperatively with antibiotics. He was also referred to an internal medicine physician for hyperglycaemia and elevated HbA1c levels, leading to the diagnosis of diabetes mellitus.

## Discussion

In this case report, we describe the ultrasonographic features of wound myiasis associated with foot gas gangrene. In the present case, myiasis was caused by larvae of the botfly *L. sericata*. Although several case reports have documented the sonographic characteristics of myiasis caused by *D. hominis* larvae, to our knowledge, a detailed description of the sonographic features of myiasis involving *L. sericata* has not yet been reported.

The common green bottle fly *L. sericata* (Meigen), formerly *Phaenicia sericata*, is a common guest of carcasses, faeces, and rubbish. *L. sericata* has important roles in forensic science, medicine, and veterinary medicine. In forensic science, larvae and maggots help determine the period of insect colonization. Insect multiplication is related to the time of death and assists detectives in their investigation [17]. *L. sericata* is one of the species commonly used for medical purposes, known as maggot therapy, and Dariusz Bazaliński et al. reported that maggot therapy with *L. sericata* is a safe and effective method for removing dead tissue during wound treatment [17,18].

POCUS is often performed for conditions associated with cutaneous myiasis. Sonographic findings of cutaneous myiasis caused by *D. hominis* larvae have been previously described as follows: the larvae appear hyperechoic with structural shapes that may be round, oval, linear, teardrop-shaped, or rod-like, depending on the orientation of the ultrasound probe. The larval structure is typically surrounded by an anechoic to hypoechoic rim and often accompanied by posterior acoustic shadowing [3,[6], [7], [8], [9], [10], [11], [12], [13], [14], [15]]. Larval movement has been observed occasionally during ultrasound examinations [11,14,15]. A cobblestone appearance, representing surrounding inflammatory changes, was also observed in some cases [10]. Some authors have reported that twinkle artefacts are observed by colour Doppler scans, representing noise within the Doppler signal caused by reflections from a highly echogenic surface [13,19].

Overall, the sonographic features observed in our case closely resembled those previously reported for cutaneous myiasis caused by *D. hominis* larvae. In our patient, *L. sericata* larvae appeared hyperechoic, with a distinct posterior acoustic shadow. The active movement of the larvae was clearly demonstrated in Supplementary 2 and 3. A cobblestone-like appearance associated with the gas was also evident. When colour Doppler imaging was applied, the larvae exhibited a twinkle artefact, which was consistently observed across a range of Doppler flow settings (10-79 cm/s). This sign proved particularly valuable for differentiating larvae from gas, as both are highly echogenic. However, gas does not produce twinkle artefacts. The Doppler flow setting also played an important role in distinguishing the structures; at 10 cm/s, the blood vessels displayed flow signals, whereas no flow was detected at 79 cm/s. Therefore, colour Doppler imaging facilitates the evaluation of structures even in the presence of overlying debris and gas. It should be noted that, as spectral Doppler assessment using either pulsed-wave or continuous-wave modalities was not performed at the time of lesion evaluation, the potential contribution of larval vascularity to the observed twinkle artefact cannot be excluded.

The presence of surrounding soft tissue oedema may have contributed to the inability to clearly visualise larval structures on CT images in our case. In addition, larval movement during image acquisition may have introduced motion-related artefacts, resulting in image blurring to some extent.

For differentiation of *L. sericata* from *D. hominis*, clusters of smaller, linear, hyperechogenic structures on ultrasound may serve as a potential imaging clue, as *L. sericata* larvae are generally smaller than those of *D. hominis* and are typically present in clusters, whereas myiasis caused by *D. hominis* usually involves a single larva.

## Conclusion

To our knowledge, this is the first comprehensive description of the ultrasonographic features of wound myiasis caused by *L. sericata* associated with foot gas gangrene. The sonographic findings, including hyperechoic larvae with posterior acoustic shadowing, a cobblestone appearance, and the presence of a twinkle artefact on colour Doppler imaging, closely resemble those reported for *D. hominis* infestation. Consistent detection of the twinkle artefact across varying Doppler flow settings proved valuable in distinguishing larvae from gas, thereby improving diagnostic confidence in a complex clinical setting. These observations emphasise the diagnostic value of POCUS, particularly in the assessment of self-propelling hyperechoic structures with posterior acoustic shadowing in combination with colour Doppler imaging, for the prompt identification of wound myiasis, even in the presence of debris and intralesional gas.

## Ethics approval

The research was conducted ethically in accordance with the World Medical Association Declaration of Helsinki.

## Availability of data and materials

The datasets generated during and/or analysed during the current study are available from the corresponding author on reasonable request.

## Authors’ contributions

YM and YT served as attending emergency physicians, while TK acted as the resident physician; all were involved in the initial management of the case in the emergency department. YM performed the ultrasound examinations and provided preliminary interpretation. JN and TT performed the emergency surgery and were responsible for the patient’s postoperative care. YO reviewed and confirmed the ultrasound findings. HA performed DNA analysis of the larval specimens and identified the species. YM drafted the manuscript and prepared figures. YM and TK conducted the literature review and developed figure and video legends. YT supervised the case report. All authors reviewed and approved the final version of the manuscript.

## Patient consent

Written informed consent was obtained from the patient and his family. The authors affirm that the patient provided informed consent for publication of the images in Figure(s) 1A, 1B, and 1C.

## Appendix Supplementary materials

Supplementary Video 1. The subcutaneous abscess was readily compressible upon the application of pressure with the ultrasound probe. Supplementary Video 2. Self-propelling hypoechoic structures were consistent with those of the larvae. Supplementary Video 3. Longitudinal sonographic profile of the larvae was observed in the subcutaneous layer. Supplementary Video 4. The larval structure was characterized by a sonographic twinkle sign, and the adjacent blood vessels showed flow signals at a Doppler setting of 10 cm/s. Supplementary Video 5. Vascular signals were absent at a Doppler velocity of 79 cm/s; however, the twinkle artefact of the larval structure persisted. Image, video 1 dummy alt text

## References

[1] Schiff T.A. (1993). Furuncular cutaneous myiasis caused by *Cuterebra* larva. J Am Acad Dermatol.

[2] McGraw T.A., Turiansky G.W. (2008). Cutaneous myiasis. J Am Acad Dermatol.

[3] Papineni V., Dieu S., Rennie W.J. (2022). The human botfly “bubbling sign”: ultrasound features of cutaneous furuncular myiasis. Indian J Radiol Imaging.

[4] Huang Y.L., Liu L., Liang H., He J., Chen J., Liang Q.W. (2020). Orbital myiasis: a case report and literature review. Medicine.

[5] Carrera K.G., Hassen G., Camacho-Leon G.P., Rossitto F., Martinez F., Debele T.K. (2022). The benefits and barriers of using point-of-care ultrasound in primary healthcare in the United States. Cureus.

[6] Richter J., Schmitt M., Müller-Stöver I., Göbels K., Häussinger D. (2008). Sonographic detection of subcutaneous fly larvae in human myiasis. J Clin Ultrasound.

[7] Quintanilla-Cedillo M.R., León-Ureña H., Contreras-Ruiz J., Arenas R. (2005). The value of Doppler ultrasound in diagnosis in 25 cases of furunculoid myiasis. Int J Dermatol.

[8] Bouer M., Rodriguez-Bandera A.I., Albizuri-Prado F., Lobos A., Gubeling W., Wortsman X. (2016). Real-time high-frequency colour Doppler ultrasound detection of cutaneous *Dermatobia hominis* myiasis. J Eur Acad Dermatol Venereol.

[9] Denofre ATAS, Silva A.C.B., Stecca C.M., Magalhães R.F., Buffo T.H. (2023). Small hole in the skin with an unexpected image in ultrasound. JAAD Case Rep.

[10] Davis C.A., Patterson J., Hampton K.A. (2022). Point-of-care ultrasound findings in a case of botfly myiasis contracted in the United States. Wilderness Environ Med.

[11] Letaïef Z., Walgraeve M.S. (2025). Use of ultrasound in the diagnosis of travel-acquired cutaneous myiasis in a pediatric patient. Cureus.

[12] Paviglianiti G., Cariello V., Vaccaro M., Pizzicato P., Minelli R., Di Rosa G. (2025). Ultrasound features of cutaneous myiasis: a rare case in a child. J Ultrasound.

[13] Jerome D., Stacey M., Newbigging J. (2023). Twinkle artifact observed during POCUS of a human myiasis caused by the *Dermatobia hominis*botfly. POCUS J.

[14] Jones C.H., Leon M., Auerbach J., Portillo-Romero J. (2020). Ultrasound detection of human botfly myiasis of the scalp: a case report. Cureus.

[15] Minakova E., Doniger S.J. (2014). Botfly larva masquerading as periorbital cellulitis: identification by point-of-care ultrasonography. Pediatr Emerg Care.

[16] Demirel-Kaya F., Orkun Ö, Çakmak A., İnkaya AÇ, Öcal M., Erguven S. (2016). A case of extensive wound myiasis caused by *Lucilia sericata* (diptera: calliphoridae) in a patient with maxillary sinus squamous cell carcinoma, in Turkey. J Arthropod-Borne Dis.

[17] Roozbehani M., Masoori L., Moradi M., Shamseddin J. (2019). Myiasis of mandible due to *Lucilia sericata*, in diabetic woman patient: a case report. Arch Clin Infect Dis.

[18] Bazaliński D., Kózka M., Karnas M., Więch P. (2019). Effectiveness of chronic wound debridement with the use of larvae of *Lucilia sericata*. J Clin Med.

[19] Rahmouni A., Bargoin R., Herment A., Bargoin N., Vasile N. (1996). Color Doppler twinkling artifact in hyperechoic regions. Radiology.

