# Supplementary figures and images for: Ultrasonographic features of Lucilia sericata-induced wound myiasis with foot gas gangrene: A case report
Source: Radiol Case Rep. 2026 Apr 11;21(7):2728–34. doi: 10.1016/j.radcr.2026.03.016 (PMC13092475; doi:10.1016/j.radcr.2026.03.016)

## Slide 1
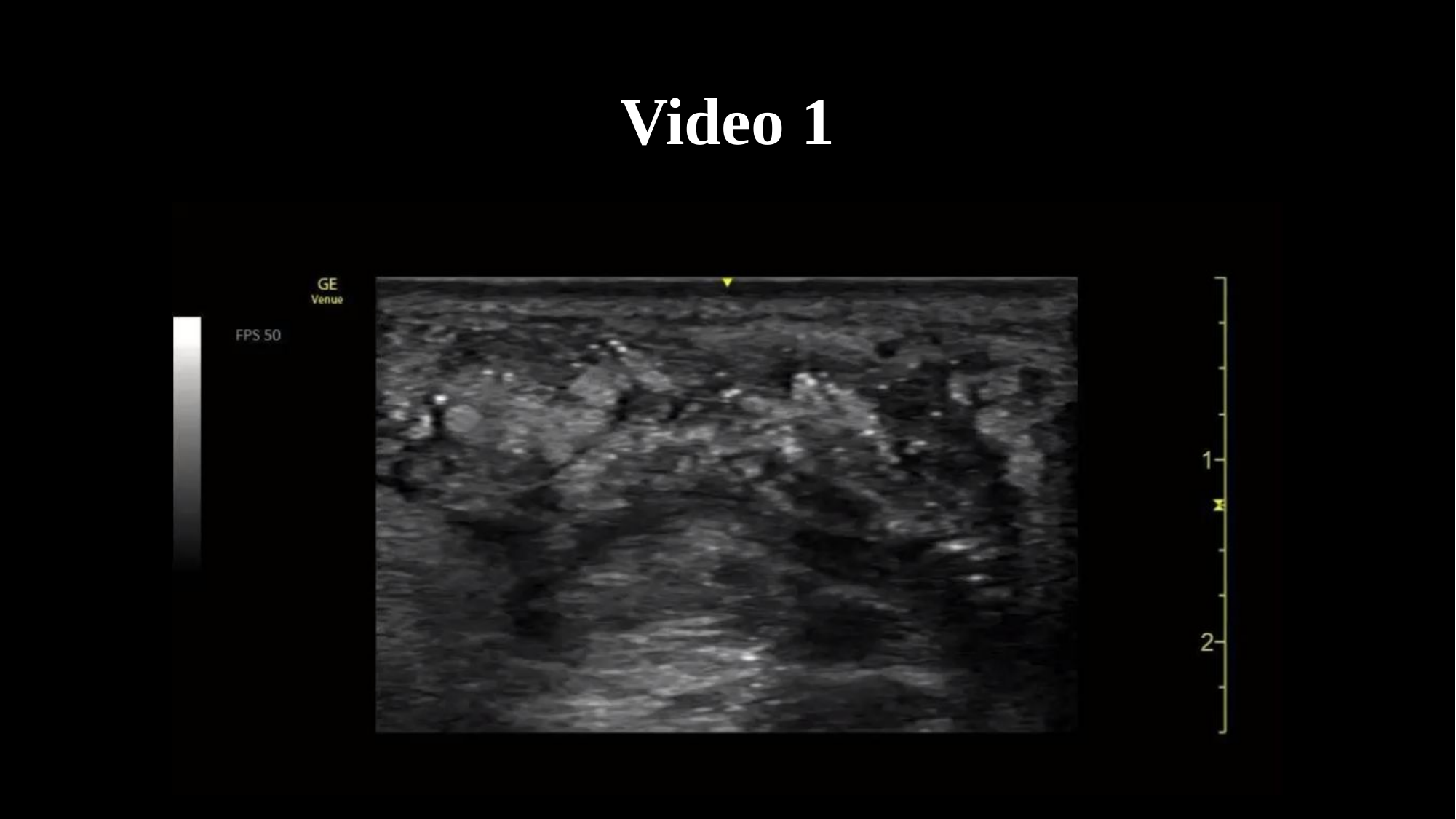

# Video 1

## Slide 2
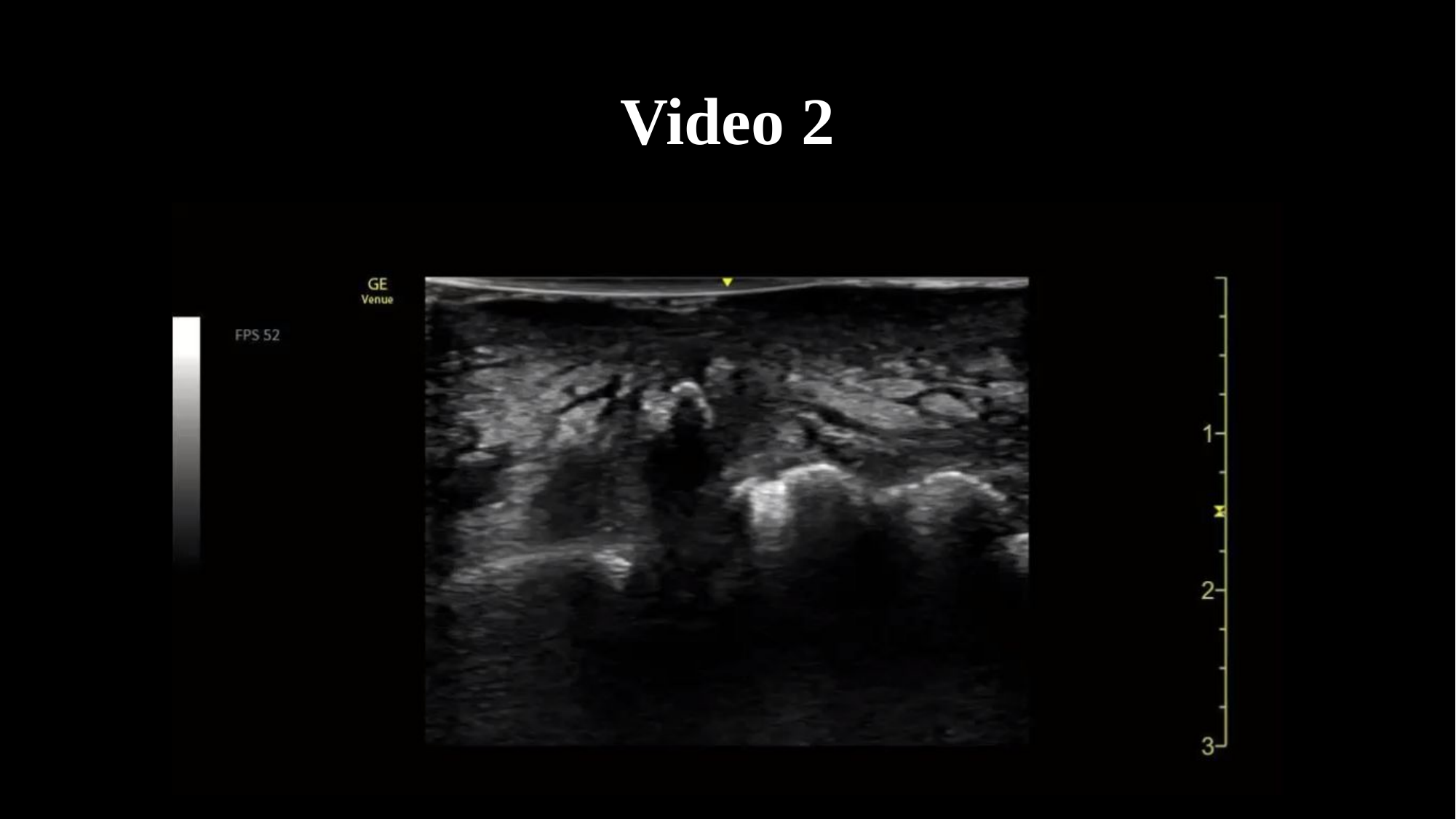

# Video 2

## Slide 3
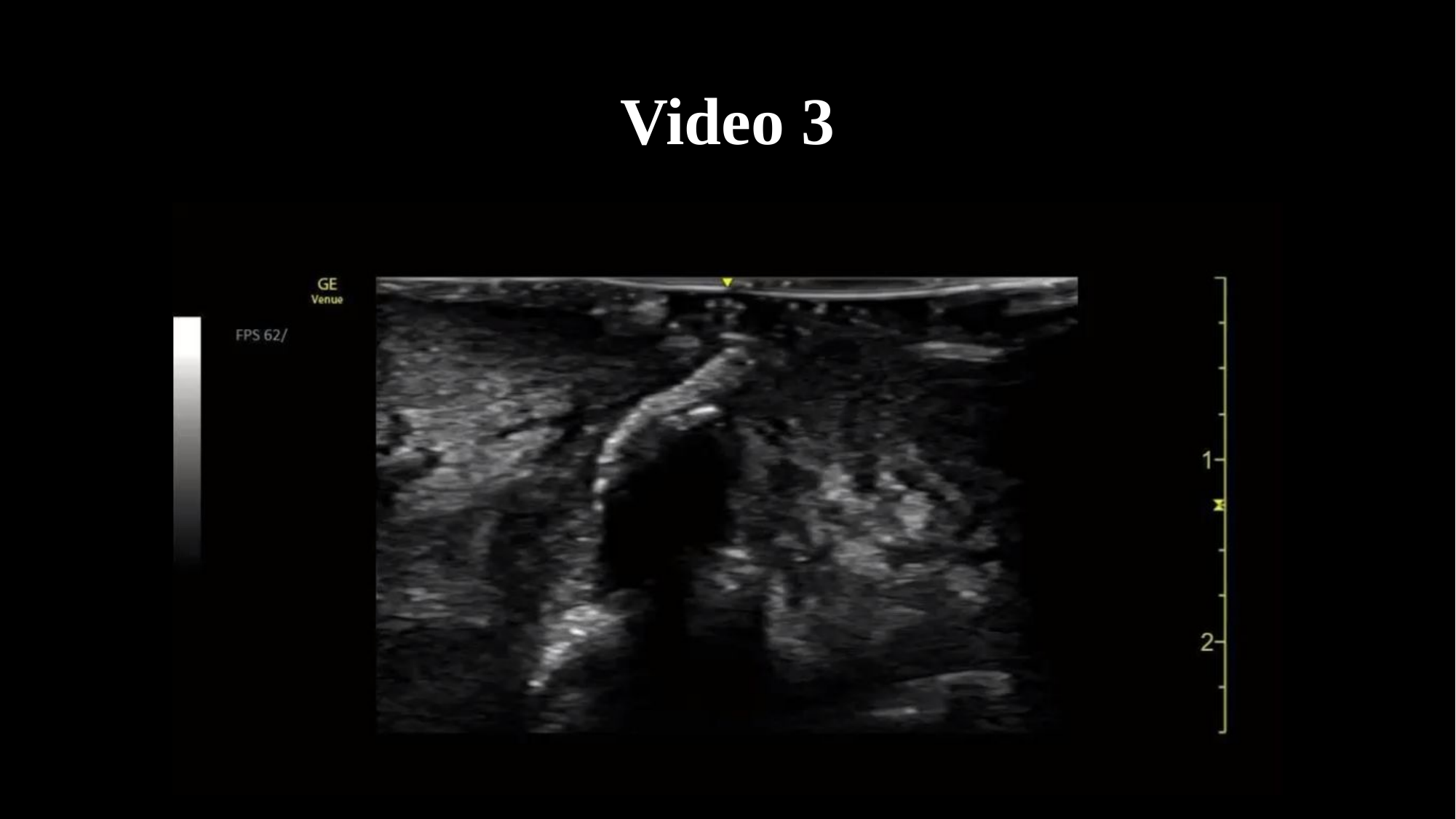

# Video 3

## Slide 4
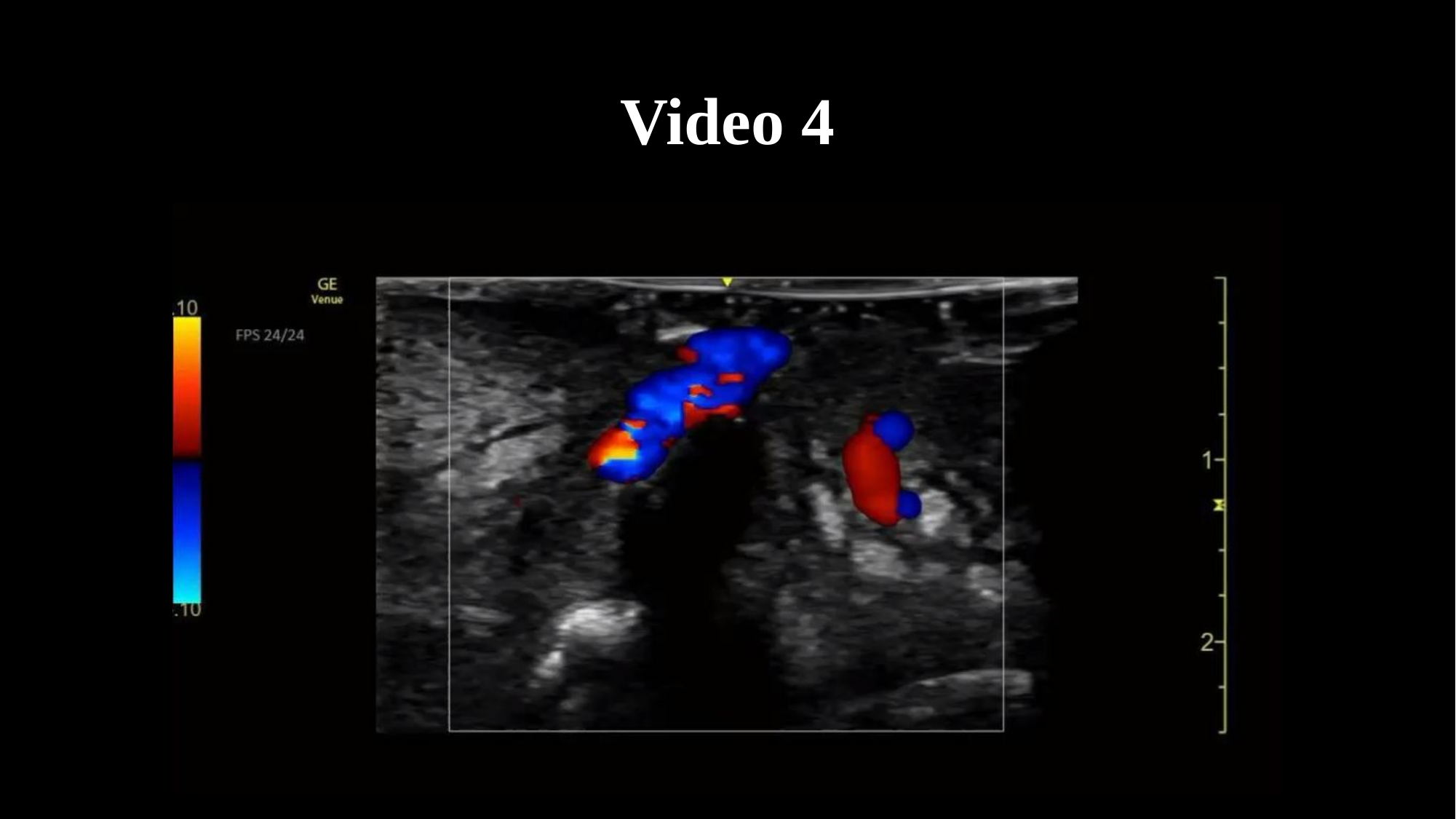

# Video 4

## Slide 5
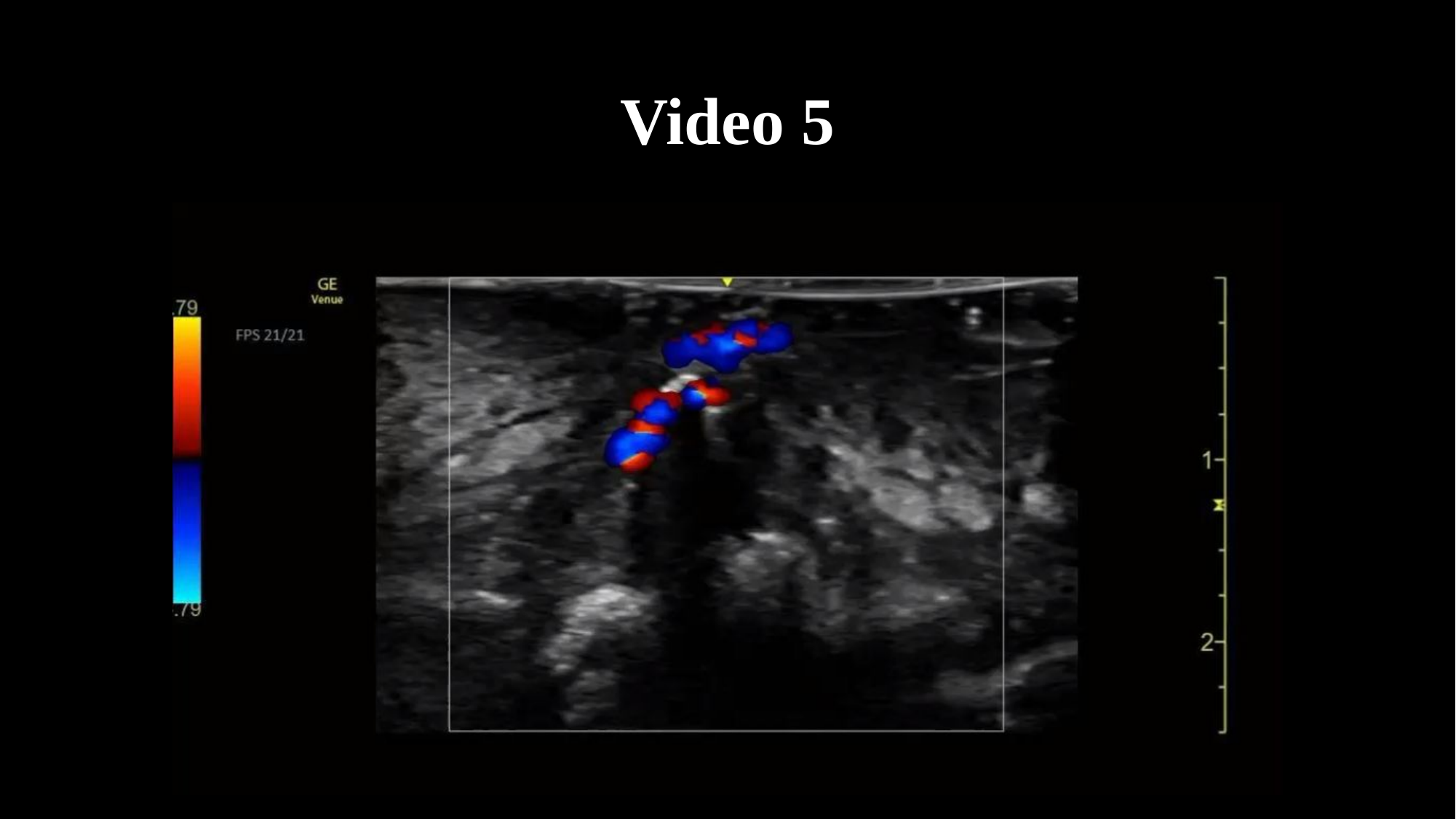

# Video 5

Supplement: Supplementary file 1 — Supplementary Video 1. The subcutaneous abscess was readily compressible upon the application of pressure with the ultrasound probe. Supplementary Video 2. Self-propelling hypoechoic structures were consistent with those of the larvae. Supplementary Video 3. Longitudinal sonographic profile of the larvae was observed in the subcutaneous layer. Supplementary Video 4. The larval structure was characterized by a sonographic twinkle sign, and the adjacent blood vessels showed flow signals at a Doppler setting of 10 cm/s. Supplementary Video 5. Vascular signals were absent at a Doppler velocity of 79 cm/s; however, the twinkle artefact of the larval structure persisted. [file mmc1.pptx]
